# Synthesis of Cellulose Hexanoate, Benzoate, and Mixed Esters: Exploring Their Potential as Enzyme Immobilization Platforms

**DOI:** 10.1002/mabi.202500221

**Published:** 2025-08-06

**Authors:** Roberta Teixeira Polez, Thamiris Voltarelli Ferracini, Samuel Filipe Cardoso de Paula, Rachel Passos de Oliveira Santos, André L.M. Porto, Elisabete Frollini

**Affiliations:** ^1^ Macromolecular Materials and Lignocellulosic Fibers Group Center for Research on Science and Technology of BioResources Sao Carlos Chemistry Institute University of Sao Paulo Sao Carlos Brazil; ^2^ Department of Bioproducts and Biosystems School of Chemical Engineering Aalto University Espoo Finland; ^3^ Organic Chemistry and Biocatalysis Group Sao Carlos Chemistry Institute University of Sao Paulo Sao Carlos Brazil; ^4^ Materials and Environmental Process Optimization Research Group Postgraduate Program in Environmental Technology University of Ribeirao Preto Ribeirao Preto Brazil

**Keywords:** cellulose esters, cellulose ester spheres, electrospun mats, enzyme immobilization, layered structure mats, racemic mixture resolution

## Abstract

This study utilizes cellulose sourced from cotton linters to synthesize cellulose esters—hexanoate, benzoate, and mixed hexanoate‐benzoate—with varying degrees of substitution (DS). These esters create electrospun mats that immobilize *Pseudomonas fluorescens lipase* (PFL), also in a configuration where an intermediate layer is added to a mat using an airbrush filled with PFL, covered by a third layer of electrospun mat. PFL‐incorporated spheres are produced from cellulose ester solutions. DS, acyl chain length, and electrospinning parameters influence the morphology of the electrospun mat, which consists of nanofibers and ultrafine fibers. The PFL‐incorporated mats show poor catalytic activity in resolving racemic (*R*,*S*)‐2‐chloro‐1‐phenylethanol, likely due to enzyme deactivation from high‐voltage electrospinning. In contrast, mat‐layered structures with PFL immobilized without voltage nearly doubled the conversion rate, although it was still lower than that of free enzymes. Spheres enhanced biocatalysis, achieving a 40% conversion rate with 94% enantiomeric purity while retaining 76% of their initial conversion rate in a subsequent reaction cycle. This research is the first to explore cellulose esters for the enzymatic immobilization of PFL to resolve a racemic mixture. The findings may enable PFL‐incorporated structures in broader biocatalysis applications; the materials created may be tested to support the immobilization of other enzymes.

## Graphical abstract

Cellulose sourced from cotton linters was utilized to synthesize cellulose esters, specifically hexanoate, benzoate, and a mixed hexanoate‐benzoate variant, which were evaluated as platforms for enzyme immobilization. These esters were shaped into various architectural forms, including electrospun mats, layered sandwich structures, and spherical shapes. This research underscores the potential of cellulose esters in developing advanced, recyclable platforms for enzyme immobilization.

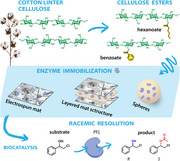

## 1 Introduction

Cellulose esters, produced by esterification of the hydroxyl groups on cellulose monomers (anhydroglucose units, AGUs), are versatile derivatives with tunable properties that make them valuable for a wide range of applications [1, 2, 3]. Previous research has greatly enhanced our understanding of ester synthesis in homogeneous media, especially through the use of the LiCl/DMAc solvent system [4, 5]. This foundational work has laid the groundwork for the synthesis of hexanoate, benzoate, and mixed esters in the current study.

A well‐established cellulose derivatization method involves using *N*,*N*‐dimethylacetamide (DMAc) with lithium chloride (LiCl) as a cosolvent, a system introduced by McCormick et al. in 1979 [6]. This system disrupts the strong hydrogen bonds within cellulose, facilitating dissolution and precise polymer modification, which is essential to achieve specific degrees of substitution (DS) and regioselectivity [7, 8]. DMAc/LiCl remains widely used for cellulose analysis, modification, and processing due to its effectiveness as a non‐derivatizing solvent system [9, 10, 11, 12, 13]. Furthermore, its alignment with sustainable practices enhances its appeal, as both DMAc and LiCl can be efficiently recovered and reused, with recovery rates exceeding 99.50% and 99.85%, respectively [14]. Although the exact dissolution mechanism in LiCl/DMAc is still debated, it is widely believed that the formation of a series of Li_x_(DMAc)_y_Cl_z_ complexes enables “free” chloride ions to disrupt hydrogen bonds and other molecular interactions, leading to solubilized cellulose chains [15, 16]. This solvent system has been used in various other applications, including the development of hydrogels [17]; Yangyang [18, 19], the creation of 3D‐printed cellulose‐based fabrics [20], and the extraction of polysaccharides from bacterial sources [21], along with other innovative uses.

Alongside the various applications of cellulose esters recognized until the end of the 1990s, the electrospinning of cellulose esters emerged as a viable option starting around the year 2000 [22]. Electrospinning stands out as an effective method for generating fibers with diameters ranging from nanometers to micrometers, high surface area‐to‐volume ratios, high porosity, and unique morphological characteristics [23, 24, 25]. These nanofibers can exhibit enhanced mechanical properties and be tailored to meet specific application requirements [26, 27, 28, 29]. A particularly promising application of these cellulose ester nanofibers is as a platform for enzyme immobilization [30, 31, 32]. The large surface area makes them ideal for developing efficient and stable biocatalytic systems [31].

Cellulose acetate membranes have demonstrated the highest enzymatic activity for immobilized catalase, alcohol oxidase, and glucose oxidase. In contrast, hydrophobic cellulose propionate and butyrate membranes provide superior storage stability but lower catalytic activity [33]. Although effective for immobilization, covalent binding methods can cause structural changes in enzymes that reduce their activity. Nevertheless, enzyme immobilization platforms remain valuable in industrial biocatalysis, biosensors, and biomedical applications, where they can enhance enzyme stability, activity, and reusability [34, 35]. Interest in industrial enzyme technology is increasing, particularly in protein engineering and enzymology in unconventional media, which expands the potential applications of enzymes as catalysts in industrial processes [36]. Enzymes, particularly lipases, are now widely used in the resolution of racemates and the synthesis of chiral drugs, agrochemicals, and pesticides with high enantiomeric purity, owing to the advantages of biocatalysts over traditional chemical routes [37, 38].

In addition to electrospun mats, cellulose esters can be shaped into spheres with high surface areas, making them ideal for batch processes and easy handling [34, 39]. Lipases immobilized in cellulose‐biopolymer spheres using ionic liquids achieved an immobilization yield of up to 52% and retained 95% of their residual activity after reuse [40].

This study explores the synthesis as processing of cellulose esters—including hexanoate (Hx), benzoate (Bz), and mixed esters (HxBz)—as novel, functional, fossil‐free materials. Electrospun nanofiber mats were produced to investigate the effects of the degree of substitution (DS) and acyl chain length on fiber morphology and diameter. These materials were further evaluated for their potential use as renewable platforms for biocatalysis—as far as is known for the first time. The direct immobilization of Pseudomonas fluorescens lipase (PFL) into electrospun fibers presented challenges, including the potential inactivation of the enzyme under high‐voltage conditions. Alternative immobilization strategies were developed to overcome these limitations: PFL‐incorporated electrospun layered mats and encapsulated spheres. The layered mats are arranged in a sandwich‐like configuration, adding the intermediate layer to an already‐formed ester mat on the collector using an airbrush filled with PFL and tetrahydrofuran—without voltage exposure—followed by electrospinning another mat layer on top. Encapsulated spheres provided a secondary option for eliminating high‐voltage processing. These ester‐based mats and spheres were evaluated for their effectiveness as enzyme immobilization platforms, with efficiency assessed through the resolution of the racemic mixture of (*R*,*S*)‐2‐chloro‐1‐phenylethanol.

## 2 Methods

### 2.1 Cellulose Dissolution and Esterification

Cellulose pulp derived from cotton linters was supplied by Nitro Química SA (São Paulo, Brazil). Before dissolution and derivatization, the cellulose underwent mercerization, an alkali pretreatment, to reduce crystallinity and enhance solubility [8]. Briefly, the cellulose sheets were cut into strips, ground in a micro‐knife mill (Marconi MA048, Piracicaba, Brazil), and immersed in 20 wt.% NaOH solution (1:50 w/v; Neon) for 1 h at 0°C [41]. After alkali treatment, the cellulose exhibited the following properties: an average molecular weight of 133 ± 0.9 kDa, a crystallinity index (CrI) of 52%, an α‐cellulose content of 92.9% ± 0.9%, and an ash content of 0.048% ± 0.007%. These properties were determined using the following methods: molecular weight via ASTM D1795‐13 with an Ostwald‐type capillary viscometer (Cannon Fenske size 150, Laborglas, São Paulo, Brazil) and cupriethylene diamine (CUEN; Merck) as a solvent [42]; CrI by X‐ray diffraction (Rigaku RINT‐2000 goniometer, Tokyo‐Japan, configured with Cu radiation (Kα = 1.542 Å) at 50 kV, 100 mA, and 2° min^−1^ speed) using the Segal method [43]; and α‐cellulose and ash content using TAPPI T 429 cm‐10 and TAPPI T 211 om‐02, respectively.

According to an established method, cellulose was dissolved in a LiCl/DMAc (5 wt.%) solvent system (Êxodo Científica/Neon) under a nitrogen atmosphere for 90 min at 160°C [44, 45]. After dissolution, esterification was performed by adding the appropriate esterifying agents—hexanoic anhydride or benzoyl chloride (Sigma Aldrich)—at molar ratios of 3, 6, and 12 reagent/AGU to achieve DS of 1, 2, and 3, respectively [46]. Hybrid esters were synthesized using a mixture of hexanoic anhydride, benzoyl chloride, and triethanolamine, with the overall molar ratio of the mixture set at three or six. The reaction was conducted at 110°C for 240 min, followed by cooling to room temperature and precipitation in methanol.

The resulting cellulose esters were designated **Hx** (cellulose hexanoate), **Bz** (cellulose benzoate), and **HxBz** (hexanoate‐benzoate hybrids), followed by the corresponding molar ratio of reagent to AGU in this section (e.g., 3, 6, or 12).

### 2.2 Ester Characterization

#### 2.2.1 Atomic Absorption Spectrophotometry (AAS)

To evaluate the potential residual lithium content in the esters and assess the efficiency of the purification process, the esters were analyzed using atomic absorption spectrophotometry (AAS) with a PerkinElmer PinAAcle 900‐T instrument (PerkinElmer, USA). An analytical standardization curve with lithium concentrations ranging from 100 to 500 ppb was prepared, yielding a linear correlation coefficient of 0.9995.

#### 2.2.2 Elemental Analysis (EA)

To assess the potential residual content of DMAc, the nitrogen content was quantified using a Flash Smart Elemental Analyzer (Thermo Scientific, Waltham, MA, USA).

#### 2.2.3 Fourier‐Transform Infrared Spectroscopy (FTIR)

The cellulose esters were analyzed using Attenuated Total Reflectance Fourier‐Transform Infrared spectroscopy (ATR‐FTIR) with a Bruker Tensor 27 FTIR spectrometer (Billerica, MA, USA). Scans were conducted in the 4000–500 cm^−1^ range with 32 scans at a resolution of 4 cm^−1^.

#### 2.2.4 Degree of Substitution (DS)

The degree of substitution of the esters was determined using Proton Nuclear Magnetic Resonance (1H NMR) spectroscopy. Spectra were acquired using an Agilent Technologies 400/54 Premium Shielded 400 MHz NMR spectrometer (Santa Clara, CA, USA) at 80°C, with 256 scans. A drop of trifluoroacetic acid was added to the 10 mg/mL DMSO‐*d_6_* solutions to shift the signals of residual water and the hydroxyl protons of the cellulose chains to a lower field.

The DS values were calculated based on the ratio of the integrals of the peaks corresponding to the protons of the alkyl group attached to the carbonyls (δ≈1.7–2.2 ppm) and the protons of the glucose rings (δ≈2.9–5.1 ppm) using Equation 1 for the cellulose hexanoate [47, 48, 49].

(1) $$DS=\frac{7\;I_{H\;methyl}}{3\;I_{H\;AGU}}\;$$

For cellulose benzoates, the DS was determined from the integrals of the peaks corresponding to the protons of the phenyl group (7.5–8.5 ppm) and the protons of the glucose rings (2.7–5.5 ppm) using Equation 2 [50, 51].

(2) $$DS=\frac{7\;I_{H\;phenyl}}{5\;I_{H\;AGU}}\;$$

### 2.3 Electrospinning Process and Mat Characterization

#### 2.3.1 Electrospinning

**Hx**, **Bz**, and **HxBz** esters were dissolved in a THF/DMAc (tetrahydrofuran/*N,N‐*dimethylacetamide) binary solvent system with a volume ratio of 65/35 (v/v) [23] at concentrations of 9 and 11 wt.% for 12 h at room temperature. This solvent system was unsuitable for electrospinning solutions of Bz and hybrid esters; therefore, trifluoroacetic acid (TFA) was used instead [52]. Electrospinning was conducted using an IME Technologies EC‐DIG electrospinning unit (Geldrop, Netherlands) combined with a pump (IME Technologies NE‐1000). Preliminary runs were conducted to test electrospinning conditions and optimize the process for producing electrospun mats. Optimization of parameters, including applied voltage (15, 20, and 25 kV), needle‐collector support distance 10 cm, and solvent flow rate (5.5, 15.5, and 45.5 µL min^−1^), was performed through testing. Electrospun mats were collected using a static collector.

#### 2.3.2 Scanning Electron Microscopy (SEM)

The microstructure of the mats and the morphology of the fibers were evaluated using SEM images taken from the surface of the mat. These images were captured using an LEO 440 ZEISS instrument (Oberkochen, Germany) at 20 kV, with secondary electron (SE1) detection at a resolution of 11 mm. Before imaging, the samples were coated with a layer of gold using a BAL‐TEC MED 020 sputter coater.

#### 2.3.3 Water Contact Angle Measurement (WCA)

The dynamic water contact angle of a 4 µL deionized water droplet on each sample surface (≈1 cm^2^) was measured at 25°C using a KSV Instruments goniometer (Helsinki, Finland) and CAM 2008 image analysis software. A sequence of 500 images was captured at 1‐s intervals, starting 1 s after the water drop made contact with the substrate.

### 2.4 Enzyme Immobilization

*Pseudomonas fluorescens* lipase (PFL) enzymes were immobilized in platforms using three different methods: electrospun mats, mat‐layered structure, and encapsulation in spheres.

#### 2.4.1 Electrospun Mats

A solution with 11 wt.% **Hx6** and 100 mg of PFL were prepared at room temperature using a THF/DMAc solvent mixture (65/35) and stirred with a magnetic stirrer for 12 h. The solution was electrospun using a setup with a 10 cm distance between the needle and collector, a voltage of 25 kV, and a solution injection flow rate of 15.5 µL min^−1^. The conditions were chosen based on the results produced in the initial stage of the study. Challenges arose during the optimization of the PFL‐incorporated solution's electrospinning process, including the potential for enzyme denaturation under voltage‐induced conditions. This was postulated after the initial results of using such a mat to resolve the racemic mixture. Thus, tests were conducted using lower voltages than those selected based on the initial electrospinning exploration. This prevented mat formation, prompting an investigation into the alternative layered mat structure.

#### 2.4.2 Mat‐layered Structure

A layered approach was tested to eliminate enzyme exposure to high‐voltage processing. First, **Hx6** (11 wt.%) was dissolved in a THF/DMAc mixture (65/35) and electrospun to form the first layer on the collector. Next, PFL dissolved in THF was sprayed onto the mat using an airbrush (Vonder, Curitiba, PR, Brazil) with a capacity of 7 mL, a needle diameter of 0.2 mm, working pressure of 25 lb in^−^^2^, supplied by a compressor (COMP1, Wimpel, São Paulo, SP, Brazil). 100 mg‐PFL was applied as a second layer. Lastly, a cellulose ester solution was electrospun on top to form a mat‐layered structure. **Hx6** was chosen based on the findings from the previous study phase.

#### 2.4.3 Spheres

An alternative enzyme immobilization method was tested, avoiding high‐voltage electrospinning altogether. In this approach, PFL was immobilized in beads using an encapsulation technique involving dropping. **Hx6** and **Hx12** cellulose esters (11 wt.%) and 100 mg of PFL were dissolved in acetone. This solution was dripped into liquid nitrogen and then transferred to water for thawing. The beads were subsequently dried at room temperature until a constant weight was achieved, a process that took ≈48 h. Initial exploration indicated that **Hx6** and **Hx12** were the most appropriate esters for forming spheres under the specified conditions.

**FIGURE 1:**
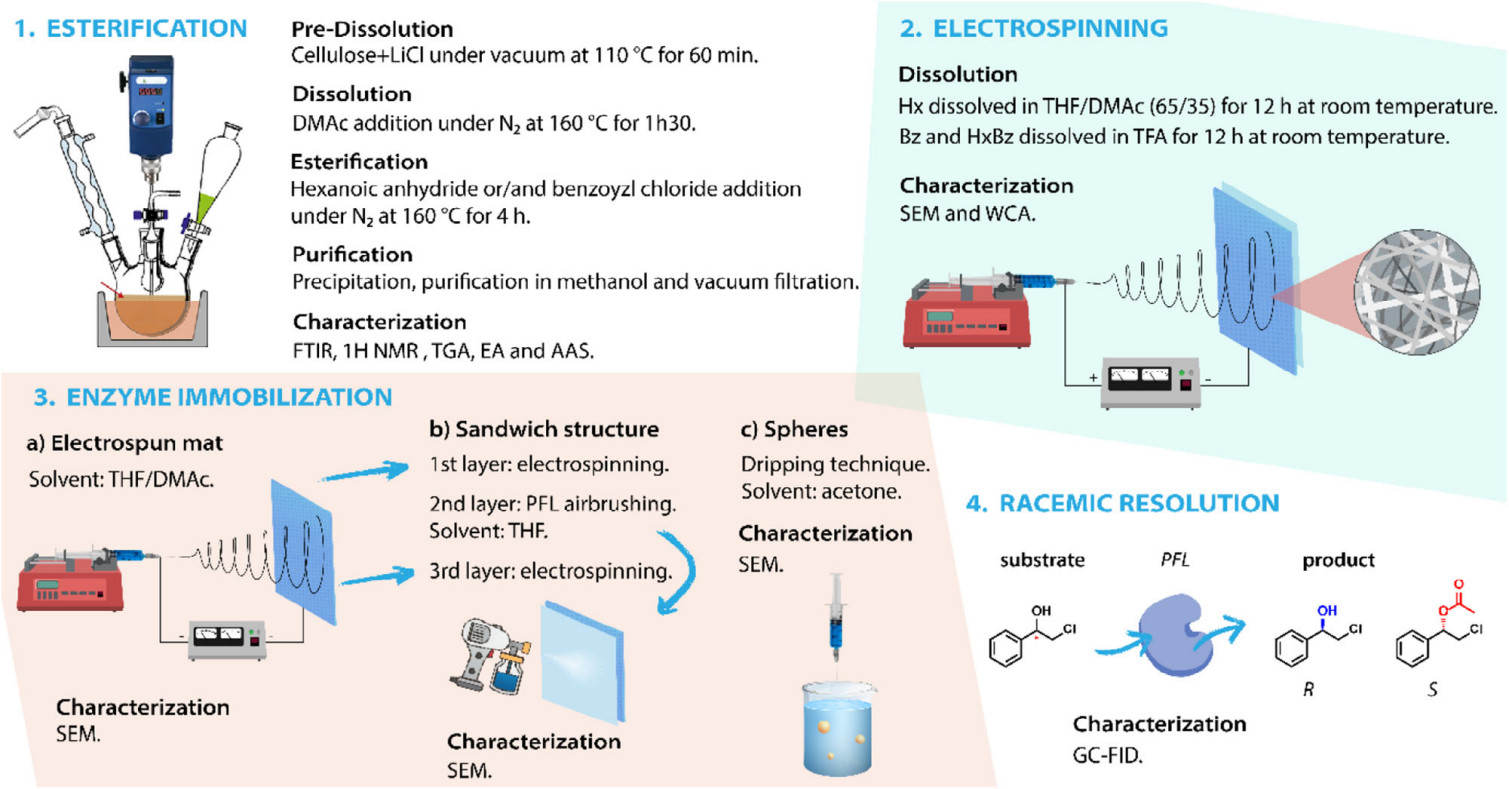
Schematic steps of the cotton linter cellulose esterification, electrospinning, enzyme immobilization, and characterization.

Figure 1 illustrates the methods employed for cellulose esterification, processing *via* electrospinning or sphere formation, enzyme immobilization, and materials characterization.

### 2.5 Kinetic Resolution of Racemic 2‐Chloro‐1‐Phenylethanol

An enzymatic kinetic resolution reaction was conducted to evaluate the enzyme immobilization. In a 10 mL vial were added 15.7 mg (0.1 mmol) of (*R*,*S*)‐2‐chloro‐1‐phenylethanol, 25 mg of the material containing immobilized lipase, 3.0 mL of *n*‐hexane, and 45 µL of vinyl acetate [53, 54]. Additionally, a higher quantity of mat‐layered structure was evaluated using 550 mg of the immobilized material. The vial was sealed with rubber septa, taped with Teflon, and kept under magnetic stirring at 130 rpm, 32°C, from 24 to 120 h.

The process was monitored every 24 h by collecting 100 µL of the reaction medium, transferring it to a 1.5 mL vial, and analyzing it with a Shimadzu GC‐2010 gas chromatograph (Kyoto, Japan). This GC was equipped with an AOC 20i auto‐injector, a flame ionization detector (FID), and a chiral column (CP‐Chiralsil‐DEX CB, 25.0 m × 0.25 mm × 0.25 µm). The GC settings were: injection volume 1.0 µL, injection temperature 250°C, split ratio 1:10, nitrogen carrier gas at 69.0 kPa, total flow 11.1 mL min^−1^, column flow 0.73 mL min^−1^, linear speed 24.1 cm s^−1^, purge flow 3.0 mL min^−1^, initial temperature 120°C for 2 min, heating rate 4°C min^−1^ for 11 min, final temperature 165°C, total analysis time 33 min, and detection temperature 270°C.

The enantiomeric excess of the product (*ee* **_p_**) and the substrate (*ee*_s_) were determined experimentally using Equations 3 and 4, where A and B represent the relative concentrations of the substrate enantiomers, and C and D represent the relative concentrations of the product enantiomers. The enantioselectivity of the process was estimated by calculating the enantiomeric ratio (*E*), as shown in Equations 5 and 6, which represents the ratio of the reaction rate constants of each enantiomer, where *c* is the conversion rate [55].

(3) $$ee_{substrate}=\frac{\left(A-B\right)}{\left(A+B\right)}\;.\;100$$

(4) $$ee_{product}=\frac{\left(C-D\right)}{\left(C+D\right)}\;.\;100$$

(5) $$E=\frac{\ln[\left(1-c\right)\left(1-ee_{product}\right)]}{\ln[\left(1-c\right)\left(1+ee_{product}\right)]}$$

(6) $$\%c=\frac{ee_{substrate}}{ee_{substrate}+ee_{product}}\;.\;100$$

A high *E* value indicates strong enantioselectivity, ensuring a high enantiomeric excess and yield. An *E* value below 10 is impractical for enantioselective processes, while values between 10 and 30 are considered good, and those above 30 are deemed excellent [55, 56].

## 3 Results and Discussion

The initial phase of this investigation involved the synthesis of cellulose esters—specifically **Hx**, **Bz**, and the hybrid **HxBz** esters—in a homogeneous medium utilizing linter cellulose as the substrate. The outcomes of these syntheses are detailed in the subsequent section.

### 3.1 Synthesis and Characterization of Cotton Cellulose Esters

To clarify the distinction of esters, from this section onward, the acronyms Hx, Bz, and HxBz will be presented with the DS values found for each molar ratio of reagent to AGU, as outlined in Table 1.

**TABLE 1:** Conditions for the derivatization of cellulose and the resulting degree of substitution (DS) for the cellulose esters.

Cellulose ester		Reagent	Ratio^a^	DS	Ester code
Hexanoate (**Hx**)	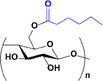	hexanoic anhydride	3	1.0	**Hx1.0**
			6	2.0	**Hx2.0**
			12	2.8	**Hx2.8**
Benzoate (**Bz**)	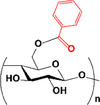	benzoyl chloride	3	0.7	**Bz0.7**
			6	1.3	**Bz1.3**
			12	1.6	**Bz1.6**
Hexanoate Benzoate (**HxBz**)	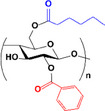	hexanoic anhydride + benzoyl chloride + triethanolamine	3	1.0	**HxBz1/1**
				1.0	
			6	0.9	**HxBz0.9/0.3**
				0.3	

^a^ Molar ratio reagent/anhydroglucose unit (AGU).

Cellulose esters were synthesized using LiCl/DMAc as the solvent system. The lithium and nitrogen content were analyzed through EA and AAS, respectively. No residual nitrogen was detected in the **Hx**‐type and **Bz0.7** samples; the other samples showed negligible amounts of nitrogen (<0.1%). No residual lithium ions were detected in the Hx samples, whereas other samples contained negligible amounts of lithium (<1 ppm) (Table S1, Supporting Information). This confirmed the washing process's effectiveness in removing the solvent system's components.

**FIGURE 2:**
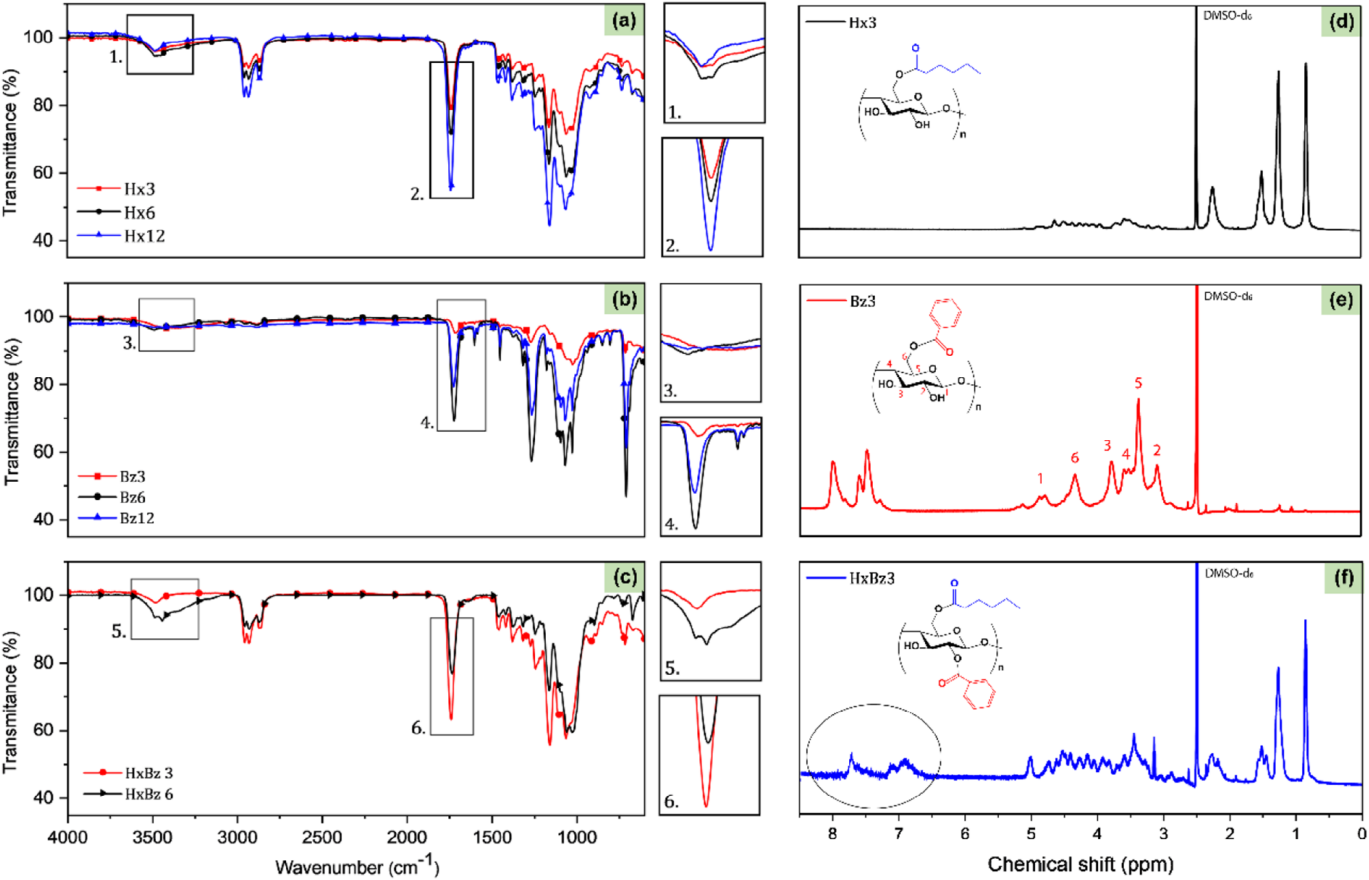
ATR‐FTIR spectra of cellulose esters with different molar ratios: (a) hexanoate, (b) benzoate, and (c) hexanoate‐benzoate. ^1^H NMR spectra of cellulose esters with different molar ratios: (d) hexanoate, (e) benzoate, and (f) hexanoate‐benzoate (range from 6.5 to 8.0 ppm, magnified for better viewing), in DMSO‐*d_6_*.

The synthesized esters were evaluated by ATR‐FTIR (Figure 2a–c). The spectra exhibited distinctive bands typical of cellulose, as noted in the literature [52], such as O‐H stretching at 3400 cm^−1^, C–H stretching at 2900 cm^−1^, CH_2_ stretching at 1370 cm^−1^; vibration of O‐C═O bond at 1240 cm^−1^; and C–O–C stretching of the glucose ring at 1040 cm^−1^ [57, 58]. As expected, the O‐H stretching band at 3400 cm^−1^ decreased, while the C═O band at 1750 cm^−1^ increased due to the formation of the ester [45, 59].

In **Bz** spectra, bands at 1630 and 1590 cm^−1^ were attributed to C═C vibrations of the aromatic ring, with additional benzene ring bands at 3060 cm^−1^ (–C–H stretching), 1430 cm^−1^ (C═C stretching), and 710 cm^−1^ (C–H out‐of‐plane bending vibration) [60, 61]. In mixed ester spectra, bands for both **Hx** and **Bz** were present, including the carbonyl band at 1750 cm^−1^, hexyl group bands at 1380, 1232, 2900 cm^−1^, and phenyl group bands at 3060, 1430, and 710 cm^−1^. These results confirmed that the modification of cellulose was accomplished.

Figure 2d‐f shows the 1H NMR spectra of synthesized cellulose esters, where the RCO‐ groups can be attached to C2‐, C3‐, and C6‐ carbons of the AGU. The peaks corresponding to the cellulose backbone appear in the δ 3.0–5.0 ppm region. In the **Hx** spectrum, the peaks corresponding to the protons of the hexyl group linked to the carbonyl are within the range of δ 1.7–2.5 ppm. The peaks of the benzene ring protons are visible between δ 7.0–8.5 ppm in **Bz** [61]. For hybrid esters, both sets of peaks were observed.

The DS of the esters was determined from their 1H NMR spectra according to Equations 1 and 2, and the results are presented in Table 1. The DS values were controlled by using the appropriate molar ratio of reagent to AGU.

The present study employed hexanoic anhydride and benzoyl chloride as an acyl donor. Acyl chlorides are recognized as potent acylating agents due to the electrophilicity of the carbonyl carbon, which is greater than that of other carboxylic acid derivatives, such as anhydrides, where the electron‐donating resonance effect of the oxygen atom diminishes the carbonyl carbon's electrophilicity. The higher electrophilicity in acyl chlorides can be attributed to the relatively weak electron‐donating effect of the chlorine atom toward the carbonyl group. However, when considering benzoyl chloride, it is important to consider the influence of the aromatic ring on the carbonyl group's reactivity, as both the steric and electronic effects of the ring can impact overall reactivity. Triethylamine was employed as a neutralization base to address the challenges posed by the hydrochloric acid generated during the reaction (Figure S1, Supporting Information). This prevents the protonation of hydroxyl groups, which would deactivate them as nucleophiles and negatively affect the reaction. Increasing the reagent/AGU molar ratio for all esters resulted in higher DS values, which aligns with trends reported in the literature [5, 57]. However, at equivalent molar ratios, the DS values for hexanoate were consistently higher than those for benzoate. For instance, at a molar ratio of 12, DS for **Hx** reached 2.8, whereas DS for **Bz** only reached 1.6 (Table 1). This suggests that hexanoyl groups can be incorporated into the cellulose chain more effectively, resulting in ester groups, compared to benzoyl groups. This difference likely stems from structural and electronic effects associated with the esterifying groups.

Hexanoyl, characterized by its flexible aliphatic chain, experiences reduced steric hindrances, favoring its accessibility and reactivity with cellulose hydroxyl sites. Since hydroxyl groups are linked to long chains, the bulky nature of the benzoyl group, characterized by its aromatic ring, can likely hinder interactions between the reactive sites, especially when the degree of substitution (DS) reaches high levels during the reaction, and available hydroxyls are increasingly reduced. Moreover, the electronic effect between the aromatic ring and the carbonyl group can further influence its reactivity. This effect was also evident in hybrid esters. For instance, at a molar ratio of 6, DS for **HxBz** reached 0.9 for hexanoate and only 0.3 for benzoate, demonstrating the preferential incorporation of hexanoyl groups. However, at a lower molar ratio (3), the DS values for hexanoate and benzoate were nearly equal, likely due to reduced competition between the esterifying groups at lower reagent concentrations. As the molar ratio increases, the steric and electronic constraints of the benzoyl group become more pronounced, further limiting its incorporation compared to the more adaptable hexanoyl group. Overall, the results suggest that hexanoate esterification is more effective in achieving a higher degree of substitution (DS) compared to benzoate, likely due to the less bulky and more flexible aliphatic structure of hexanoate, which faces fewer spatial constraints during substitution.

### 3.2 Electrospinning and Mat Characterization

The subsequent phase after synthesis involved the electrospinning of solutions containing the synthesized esters, as outlined in the following section. The esters **Hx1.0**, **Hx2.0**, **Bz1.6**, **HxBz1/1**, and **HxBz0.9/0.3** have been selected for this study phase. Table 2 summarizes the electrospinning conditions that resulted in mats made from cellulose esters. THF was chosen as a primary solvent due to its favorable properties: low surface tension (26.4 mN m^−1^), low boiling point (66°C), and low dielectric constant (7.6). Its combination with DMAc, a polar solvent, enhanced the dissolution of more polar and hydrophilic polymers, such as cellulose derivatives, ensuring better polymer solubility and fiber formation [62, 63].

**TABLE 2:** Summary of the electrospinning parameters that resulted in ester fibers, including the concentration, solvent, voltage, flow rate, and diameter of the electrospun fibers.

Entry	Ester code	Ester [wt%]	Solvent system	Voltage [kV]	Flow [µL min^−1^]	Diameter range (nm)	Diameter average (nm)	Fiber type^a^	Water contact angle (°)
1	**Hx1.0**	9	THF/DMAc	15	5.5	80–300	168	UFF	132 ± 6
2	**Hx1.0**	9	THF/DMAc	15	15.5	85–330	158	UFF	131 ± 7
3	**Hx1.0**	9	THF/DMAc	20	5.5	70–300	166	UFF	104 ± 13
4	**Hx1.0**	9	THF/DMAc	20	15.5	60–260	137	UFF	97 ± 14
5	**Hx2.0**	11	THF/DMAc	15	15.5	85–330	158	UFF	133 ± 2
6	**Hx2.0**	11	THF/DMAc	15	45.5	55–230	117	UFF	140 ± 1
7	**Hx2.0**	11	THF/DMAc	20	5.5	30–300	111	UFF	136 ± 1
8	**Hx2.0**	11	THF/DMAc	20	15.5	40–280	122	UFF	139 ± 1
9	**Hx2.0**	11	THF/DMAc	20	45.5	35–220	112	UFF	141 ± 2
10	**Hx2.0**	11	THF/DMAc	25	5.5	50–280	141	UFF	127 ± 2
11	**Hx2.0**	11	THF/DMAc	25	15.5	40–250	117	UFF	142 ± 1
12	**Hx2.0**	11	THF/DMAc	25	45.5	60–280	130	UFF	128 ± 2
13	**Bz1.6**	9	TFA	20	5.5	40–400	151	UFF	68 ± 15
14	**Bz1.6**	9	TFA	20	15.5	35–450	150	UFF	50 ± 9
15	**Bz1.6**	9	TFA	25	5.5	65–400	138	UFF	94 ± 12
16	**Bz1.6**	9	TFA	25	15.5	95–460	228	UFF	59 ± 12
17	**HxBz1/1**	9	TFA	20	5.5	30–150	77	NF	125 ± 1
18	**HxBz1/1**	9	TFA	20	15.5	45–150	85	NF	123 ± 1
19	**HxBz1/1**	9	TFA	25	5.5	30–190	78	NF	122 ± 1
20	**HxBz0.9/0.3**	9	TFA	20	5.5	35–130	65	NF	132 ± 1
21	**HxBz0.9/0.3**	9	TFA	25	15.5	40–180	75	NF	134 ± 1

^a^ NF: nanofibers (Ø ≤ 100 nm), UFF: ultrafine fibers (100 > Ø > 1000 nm).

While the THF/DMAc system was adequate for cellulose hexanoates, it proved inadequate for electrospinning **Bz** and hybrid esters. For these derivatives, TFA was used as an alternative solvent due to its properties, including effective polymer dissolution, high volatility (boiling point 72.4°C), low surface tension (17.6 mN m^−1^), and low dielectric constant (8.5) [64, 65, 66].

**FIGURE 3:**
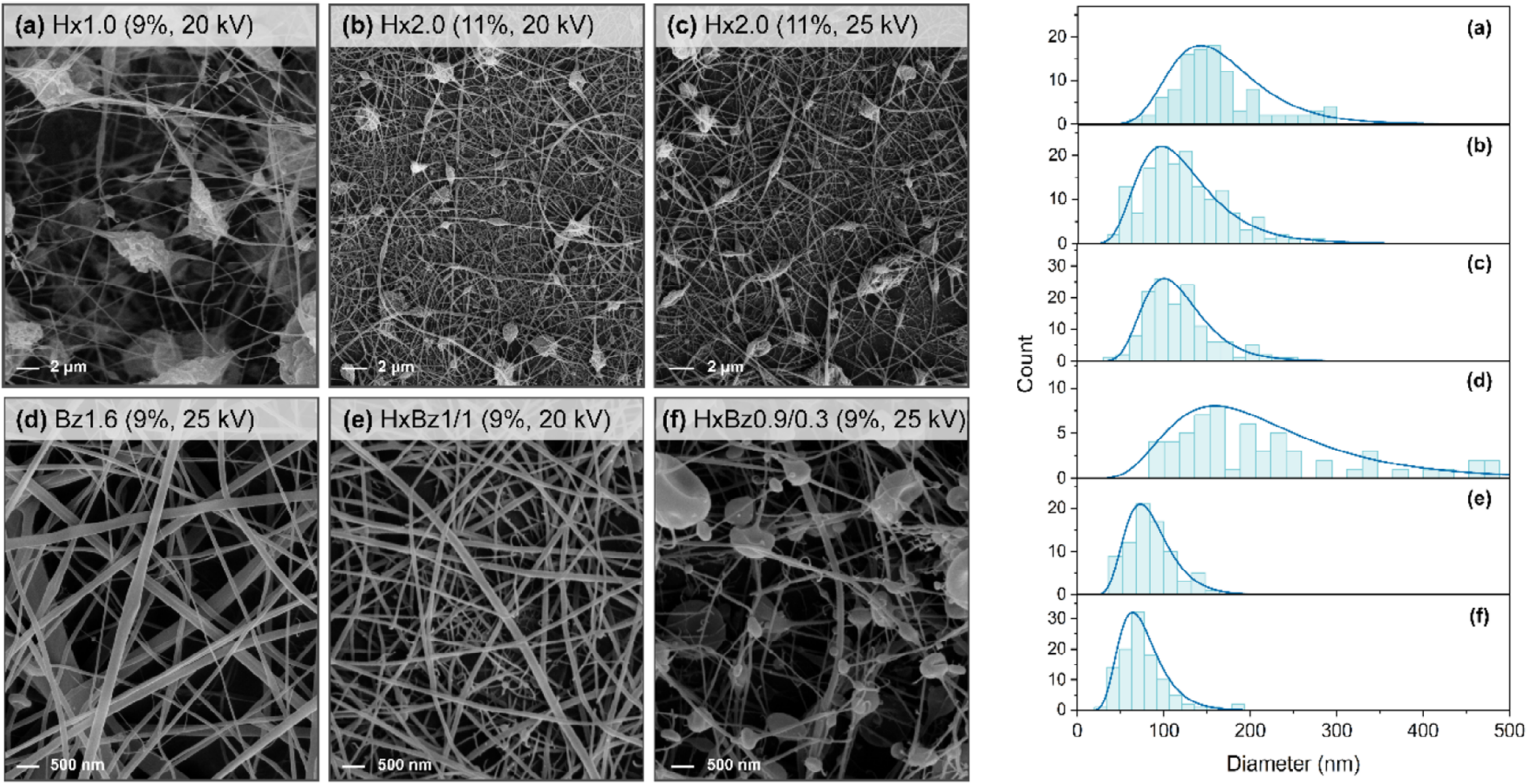
SEM images of the electrospun mats formed at a flow rate of 15.5 µL min^−1^ and their respective fiber diameter distributions. (a) Hx1.0 (9 wt% in THF/DMAc) at 20 kV. (b) Hx2.0 (11 wt.% in THF/DMAc) at 20 kV. (c) Hx2.0 (11 wt.% in THF/DMAc) at 25 kV. (d) Bz1.6 (9 wt.% in TFA) at 25 kV. (e) HxBz1/1 (9 wt.% in TFA) at 20 kV. (f) HxBz0.9/0.3 (9 wt.% in TFA) at 25 kV. Histograms do not take into consideration bead size.

Figure 3 displays SEM images of the electrospun mats, along with the electrospinning parameters that enabled their formation, accompanied by the corresponding histograms of fiber diameter distributions.

The electrospun mats obtained from **Hx1.0** (9 wt.%, Table 1) presented ultrafine fibers with diameters ranging from 137–168 nm and many beads scattered along their length (Figure 3a, **entries 1–4**, Table 2). This bead formation is often associated with low polymer concentrations, where insufficient viscosity can destabilize the fiber jet during electrospinning [65]. Other factors, such as surface tension and net charge density, can also contribute to that phenomenon [67]. The low amount of fibers from **Hx1.0** observed likely resulted from the polymer concentration being below the entanglement concentration, which limits the chain entanglement required to form continuous fibers.

Depending on the voltage, the electric field applied during electrospinning may interact with the polar groups in cellulose esters, i.e., the hydroxyl and carbonyl groups. This interaction may lead to the alignment of polymer chains. Additionally, dipole‐dipole interactions with solvent molecules can facilitate the formation of a conductive pathway. It can be proposed that the pendant ester groups interact more significantly than the hydroxyl groups. Consequently, a higher DS may result in stronger interactions. Furthermore, a higher DS indicates a more uniform distribution of ester groups within the rings of the same chain and among different chains. This uniformity may favor the alignment of segments from neighboring chains and facilitate the formation of fibers. Along with the aforementioned factors, the flow rate also influences the properties of the electrospun material. Electrospun fibers of **Hx2.0** (11 wt.%) obtained varying the flow rate from 5.5 to 45.5 µL min^−1^ at 15 and 20 kV (**entries 7–9**, Table 2) showed that a higher flow rate promoted the formation of smaller‐diameter fibers with fewer beads. Specifically, at the highest flow rate (45.5 µL min^−1^), the fibers averaged 117 nm in diameter, ranging from 55 to 230 nm, while the lower flow rate produced fibers with an average diameter of 158 nm, ranging from 85 to 330 nm (**entries 5–6**, Table 2). A higher flow rate enhances the shear forces acting on the polymer solution as it is ejected, helping to stretch the solution jet and produce thinner fibers. It also decreases the time the polymer solution jet remains in the air, minimizing the likelihood of solidification before it is fully stretched and reducing the chances of bead formation. Additionally, higher flow rates can increase the charge density on the surface of the fibers, promoting better elongation and alignment of the polymer chains (Ahmadi [68, 69]). Other studies, such as those on cellulose hexanoate in TFA, observed a similar dependence of fiber diameter on flow rate [45].

The applied voltage directly influences the charge carried by the electrospinning jet, affecting the magnitude of electrostatic repulsion between charges and the strength of interactions between the jet and the external electric field [70]. A higher voltage on **Hx2.0** solutions favored the formation of thinner fibers (**entries 5, 8, 11,** Table 2), as indicated by the decrease in the average fiber diameter from 158 nm at 15 kV to 117 nm at 25 kV. This change also resulted in a narrower fiber diameter range, shifting from 85–330 nm at 15 kV to 40–250 nm at 25 kV, which is consistent with the findings of other studies [45]. However, higher applied voltages lead to more fluid ejection, resulting in thicker fibers (**entries 6, 9, 12,** Table 2). For example, as the voltage increased from 15 to 20 kV and then to 25 kV, the corresponding average fiber diameters were measured at 117, 112, and 130 nm, respectively. This suggests that although higher voltages generally produce finer fibers, there may be exceptions based on the concentration and fluid dynamics during electrospinning [71].

The THF/DMAc solvent produced fibers with a bead‐on‐string morphology, while TFA yielded smooth, defect‐free fibers. Beads in electrospun fibers form due to the rapid evaporation of the solvent, which is more pronounced in THF, given its lower boiling point (66°C) compared to TFA (72.4°C). Although DMAc (165°C) has a significantly higher boiling point, the dominant contribution of THF in the 65/35 THF/DMAc mixture leads to rapid solvent loss during the electrospinning process. This rapid evaporation induces phase separation within the polymer jet, resulting in regions rich in polymer and others that are poor, ultimately leading to instability in the jet [72]. As the jet stretches and cools, water vapor can condense on its surface, forming droplets that hinder its solidification [73]. These factors result in a beaded structure, where the beads represent regions of higher viscosity that do not stretch as much as the intervening polymer‐rich areas [74].

The Hx ester exhibits higher water contact angles (97°–142°), indicating greater hydrophobicity, while the Bz ester shows significantly lower angles (50°–94°), suggesting that Bz‐based fibers are more hydrophilic. An increase in voltage generally results in lower contact angles for **Hx1.0** fibers (**entries 1–4,** Table 2). For instance, at 20 kV, the WCA decreases significantly compared to 15 kV, likely due to changes in fiber morphology. The nature of the ester side groups plays a key role in this behavior. With its long, non‐polar alkyl chain, the hexanoate side group repels water, increasing hydrophobicity and resulting in higher contact angles. This suggests that the hexanoate chains are oriented on the fiber's outer surface, while the more hydrophilic cellulose backbone faces inward, amplifying the surface's water‐repellent properties. Similar studies have reported WCA of 103°–114° for cellulose ester films with long alkyl side chains, which also serve as effective barriers to water vapor transport [75, 76]. In contrast, the planarity of the benzoate group may have favored the orientation of the polar carbonyl groups toward the surface of the fiber. Additionally, the contribution from the aromatic π electron density may have further reduced the contact angle compared to the hexanoates, thereby imparting a degree of hydrophilicity to the material.

In addition to the side‐group chemical structure, the DS can influence surface wettability. At DS 1, cellulose hexanoate exhibited WCA ranging from 97° to 132°, showing notable variability. This suggests that while esterification increases hydrophobicity, some hydroxyl groups remain available for hydrogen bonding with water, leading to heterogeneous wetting behavior. However, at DS 2, contact angles were consistently higher (127°–142°) with significantly reduced variability, indicating a more uniform hydrophobic surface. This trend confirms that increasing DS enhances hydrophobicity by replacing hydroxyl groups with hydrophobic ester moieties. However, it is important to note that the observed differences in contact angle are not statistically significant (*p* > 0.05).

### 3.3 Sphere Characterization

**FIGURE 4:**
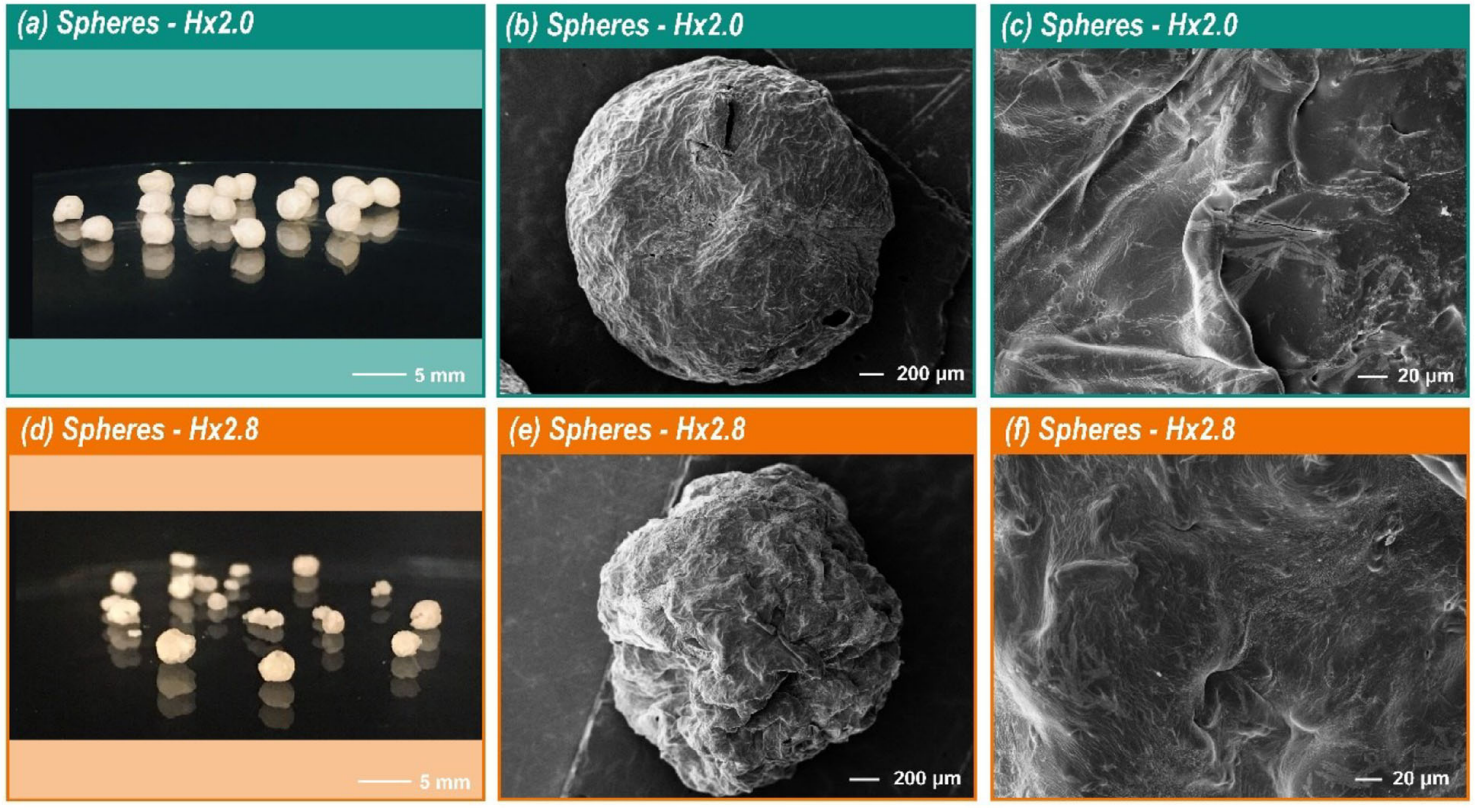
Spheres of **Hx2.0** (Ø 3.1 ± 0.3 mm) dried: photo (a) and SEM images (b, c) at different magnifications (100**×** and 1000**×**). Spheres of **Hx2.8** (Ø 2.4 ± 0.2 mm) dried: photo (d) and SEM images (e, f) at different magnifications (100× and 1000×).

In light of the results discussed later regarding the application of electrospun mats, spherical structures were also produced from cellulose ester solutions (Figure 4). The primary reason for choosing spheres was that their formation process does not require the application of voltage.

SEM images of **Hx2.0** and **Hx2.8** spheres (Figure 4b,e) revealed notable differences in size and surface morphology. The **Hx2.8** spheres were smaller (Ø 2.4 ± 0.2 mm) with a rougher surface, while the **Hx2.0** spheres were larger (Ø 3.1 ± 0.3 mm) with a smoother surface. These differences are primarily attributed to the different DS. A higher DS, as in **Hx2.8**, introduces more hydrophobic acyl groups along the cellulose backbone, which can affect the solution properties—such as viscosity, chain interactions, and surface tension—during sphere formation. These factors, in turn, influence the droplet breakup dynamics, shrinkage behavior, and final morphology. Thus, the DS is a key parameter that influences the physicochemical properties of the spheres, governing both their size and surface morphology.

### 3.4 Enzyme Immobilization and Racemic Resolution

In this phase of the study, the focus was on evaluating the potential of electrospun mats (**Hx2.0**) and spheres (**Hx2.0** and **Hx 2.8**, Table 1) as renewable platforms for biocatalysis, with *Pseudomonas fluorescens* lipase (PFL) immobilized on these structures. Benzoates and hybrid esters were excluded from this study phase because their dissolution involves TFA, which degrades the enzyme and compromises its activity.

**Hx2.0** was selected for electrospun mat preparation as it enabled the formation of nano/ultrafine fibers incorporating the enzyme. However, direct PFL incorporation into electrospun fibers posed challenges related to uniformity, stability, and possible enzyme denaturation under high‐voltage conditions. To address these issues, an alternative mat‐layered structure was prepared, consisting of an electrospun ester layer, an airbrushed PFL layer, and a second electrospun ester layer, allowing enzyme deposition while reducing its exposure to electrospinning conditions.

Furthermore, spheres prepared from **Hx2.0** and **Hx2.8** were also used as substrates for enzyme immobilization to compare with mats, since sphere formation does not require voltage application. This allowed the evaluation of enzyme performance under different processing conditions.

**FIGURE 5:**
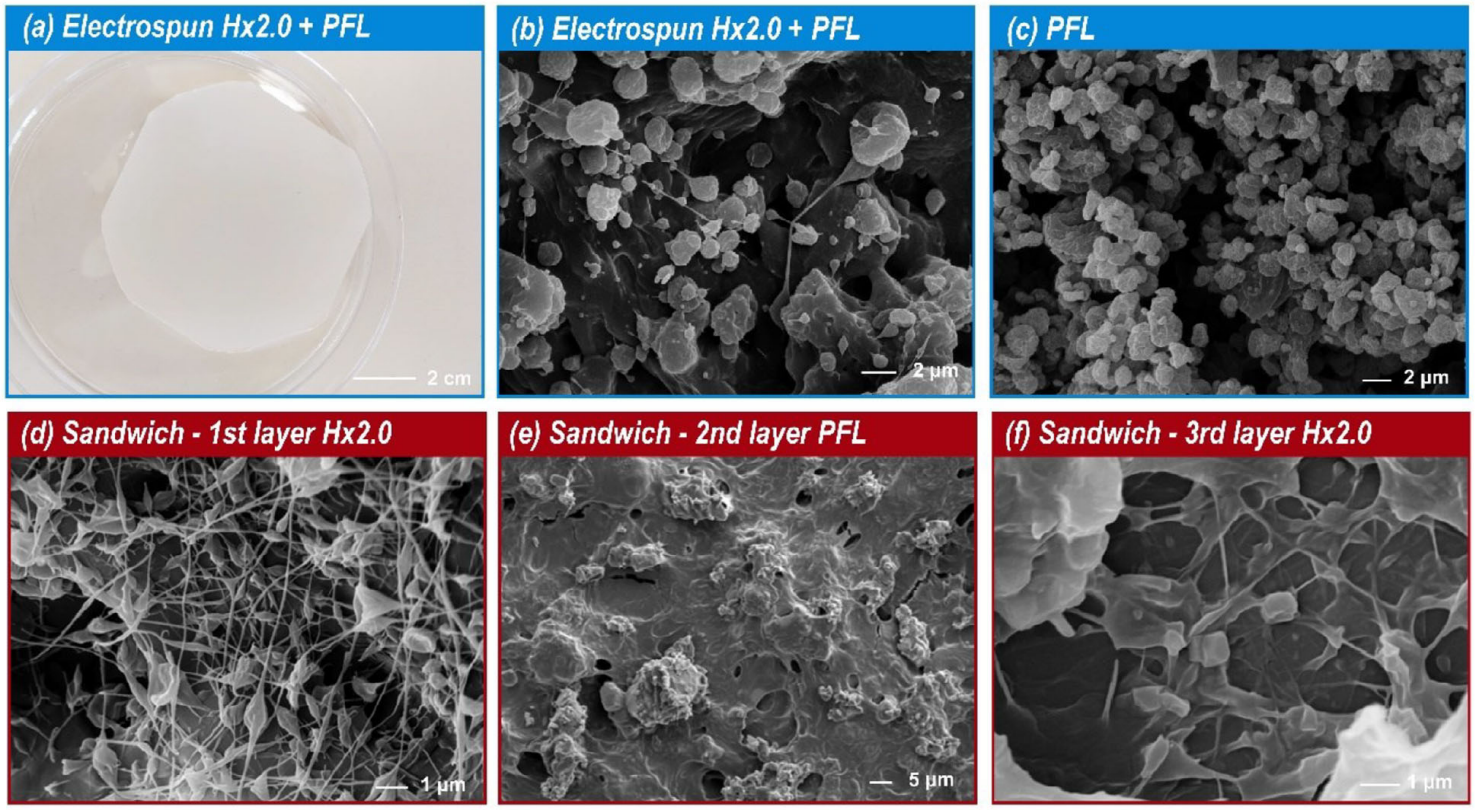
Electrospun mat of **Hx2.0** with *Pseudomonas fluorescens* Lipase (PFL): photo (a) and SEM image (b). SEM image of PFL (c). Mat‐layered structure of **Hx2.0**: SEM images of first layer electrospun **Hx2.0** (d), second layer airbrushed PFL (e), and third layer electrospun **Hx2.0** (f).

Figure 5 shows photos and SEM images illustrating the morphology and structure of the immobilization platforms. Electrospun mats of **Hx2.0** with immobilized enzyme (Figure 5a) appeared as a white, homogeneous film. However, SEM images (Figure 5b) revealed a heterogeneous structure comprising a mixture of fibers and enzyme aggregates, with enzyme morphology clearly visible (Figure 5c). The mat‐layered structures (Figure 5d,f) displayed fibers with bead‐like features, and the airbrushed PFL layer formed a cast‐like film over the fibers, as depicted in Figure 5e.

The efficiency of enzyme immobilization was assessed via enzymatic kinetic resolution of (*R*,*S*)‐2‐chloro‐1‐phenylethanol. Distinct retention times for enantiomeric pairs were observed for both racemic 2‐chloro‐1‐phenylethanol and its racemic acetylated derivative, allowing these chromatograms to serve as references for preliminary enzymatic kinetic resolution studies (Figure S2, Supporting Information). The effects of different immobilization platforms were analyzed, as shown in Table 3.

**TABLE 3:** Kinetic resolution of (*R*,*S*)‐2‐chloro‐1‐phenylethanol by a lipase from *Pseudomonas fluorescens* immobilized on mats, mat‐layered structure, and spheres.

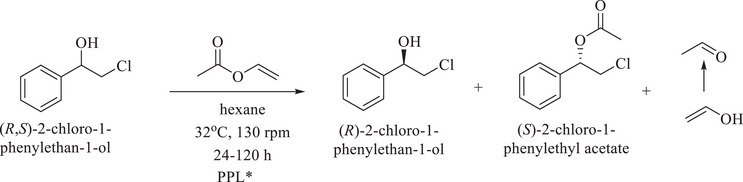						
Entry	Sample	Time [h]	*ee*_s_ [%]^a^	*ee*_p_ [%]^b^	*c* [%]^c^	*E* ^d^
1	Lipase (PFL) [87]	24	36	98	28	>100
		48	53	98	35	>100
2	Mat **Hx2.0**	24	0	0	43	—
		48	1	2	24	
3	Mat‐layered structure **Hx2.0**	24	0	3	13	—
		48	1	3	23	
4	Mat‐layered structure **Hx2.0** (550 mg)	24	2	16	9	—
		48	1	15	7	
5	Spheres **Hx2.0**	24	18	98	16	>100
		48	34	96	26	72
		96	38	93	29	42
		120	36	92	28	35
6	Spheres **Hx2.8**	24	15	98	13	>100
		48	33	96	26	66
		96	60	95	39	68
		120	64	92	40	66
**7**	Spheres **Hx2.8** Recycle	24	4	50	7	<30
		48	8	70	7	<30
		96	12	81	13	<30
		120	16	85	16	<30

^*^ PFL‐free and immobilized.

^a^ substrate enantiomeric excess.

^b^ product enantiomeric excess.

^c^ conversion.

^d^ Selectivity (Enantiomeric ratio).

Using non‐immobilized PFL, the reaction yielded a moderate conversion (c = 35%) and an excellent excess enantiomeric excess (*ee*_p_ = 98%) for (*S*)‐2‐chloro‐1‐phenylethanol acetate after 48 h, with an excellent enantiomeric ratio (*E* = 168) (**entry 1,** Table 3). The *E*‐value, which reflects enzymatic selectivity, is crucial for assessing enantiomeric excess and yield. Values below 10 render the process unfeasible, values above 10 allow efficient resolutions, and values above 30 are considered excellent [77, 78].

The use of immobilized lipase in **Hx2.0** mats, however, resulted in low conversion rates (43%, 24%, and 1%) and poor enantioselectivity (*E* < 10), indicating an unfeasible reaction (**entry 2,** Table 3). The free enzyme was more efficient when changing the substrate into the product than the enzyme in **Hx2.0** mats. These mats were produced via electrospinning under specific conditions: 10 cm distance, 25 kV voltage, and a 15.5 µm min^−1^ injection flow rate. High voltage during electrospinning can significantly impair enzyme activity, potentially leading to partial or complete inactivation [79]. This phenomenon occurs because the high electric field can induce structural changes or potential denaturation of the enzyme, similar to what is observed in pulsed electric field (PEF) applications. PEF is commonly used as a non‐thermal food preservation method to inactivate enzymes and eliminate microorganisms by applying high‐voltage electric pulses for microseconds to milliseconds [80]. This process induces electroporation, which disrupts cell membranes and can degrade enzyme activity [3].

For instance, research has shown that lipase activity can be reduced by up to 90% under PEF conditions, specifically at 88 kV cm^−1^ voltage, 2 µs pulse width, 2 s treatment duration, 0.5 Hz frequency, and a maximum temperature of 20°C [81]. Another study reported varying levels of lipase inactivation, with 62% inactivation in batch processing and 13% in continuous flow processing of ultrafiltered milk under PEF conditions (27.4–37.3 kV cm^−1^ voltage, 4 µs pulse width, 314.5 µs treatment duration, 80–100 pulses, and a maximum temperature of 34°C) [82].

Electrospinning has been studied to produce enzyme‐immobilized mats with varying effects on enzyme activity. For example, co‐electrospinning of α‐amylase with Alq3 in carbotane polymer at 19.5 kV voltage, 15 cm distance, and 0.9 mL h^−1^ flow rate resulted in the enzyme retaining 30% of its initial activity after 10 cycles [83]. In another study, Candida rugosa lipase immobilized in polyvinyl alcohol (PVA) via electrospinning at 10 kV and 17.78 cm distance showed no loss in catalytic activity compared to the free enzyme, indicating that certain conditions can preserve enzyme function [3]. These findings suggest that while electrospinning can effectively immobilize enzymes, high‐voltage settings may lead to enzyme inactivation unless carefully optimized.

To confirm whether the applied voltage would be responsible for the enzyme inactivity, numerous tests were performed to create mats using voltages lower than those specified in Table 2; however, mats did not form at the reduced voltage levels that were tested, which made it impossible to investigate whether the high voltage was leading to the negative results. Another option was then explored: a PFL‐incorporated electrospun layered mat.

An intermediate layer was added to an ester mat already formed on the collector using an airbrush filled with PFL and tetrahydrofuran—without exposure to voltage—followed by electrospinning another mat layer on top. Despite this, the use of the sandwich‐like structure as a platform resulted in reactions with low selectivity (*E* < 5), achieving only 18% conversion after 168 h (**entries 3–4,** Table 3). The mat‐layered structure improved *ee*_p_ and *E* but remained inferior to the free enzyme. In this case, the access of the racemic mixture to the enzymes may have been hindered by the type of structure used to immobilize the enzyme, as the layer on top needed to be traversed to reach the enzymes.

In addition to the issues mentioned, the solvent system used to dissolve the lipase and cellulose esters—DMAc/THF—could have adversely affected the enzymatic activity. Polar aprotic solvents such as DMAc can disrupt the network of intramolecular forces—such as hydrogen bonding, electrostatic interactions, and hydrophobic effects—that stabilize the enzyme's native tertiary structure. This disruption can lead to partial unfolding of the enzyme, compromising its conformation and catalytic function. Recent findings further support this, showing that tertiary amides such as DMAc, NMP, and DMF can diminish lipase activity [84]. Moreover, hydrophobic solvents like 1,4‐dioxan, THF, and higher alcohols are denaturants, causing inactivation at concentrations as low as 10%–30% volume as compared to hydrophilic solvents like glycerol, ethylene glycol, which can be used at concentrations of 50%–60% volume [85].

The results of this study on enzyme immobilization on electrospun mats, along with the cited literature, indicated that enzymatic activity is highly dependent on process parameters and the surrounding environment. In this context, other immobilization methods were tested, including using a different solvent (e.g., acetone) and preparing spheres of **Hx2.0** and **Hx2.8**. Free‐form lipase showed higher conversion rates compared to immobilized lipase, except for the first cycle of the **Hx2.8** spheres, which showed a higher conversion after 120 h (*c* = 40%) and a good enantiomeric ratio (*E* = 66) (**entry 6,** Table 3). Both free and immobilized lipase showed high enantiomeric purity, with 98% *ee*_p_ and 94% *ee*_p_, respectively. Similarly, research using spheres made from silk fibroin and alginate to immobilize Amano AK lipase showed a significant improvement in enantioselectivity, increasing from *E* > 100 to *E* > 200 compared to the free enzyme [86].

After the kinetic racemic resolution, **Hx2.8** spheres were recovered by filtration, washed with hexane, and reused in a second reaction cycle. Considering the significantly high enantiomeric conversion rate of the **Hx2.8** spheres combined with the possibility of recovering the material by filtration, these factors justify the reason why only this material was considered in testing the recycling procedure. The recycling of enzymes is important in biocatalytic processes. Therefore, the reutilization of the immobilized PFL on spheres was tested. In the second cycle, conversion decreased from 40% to 16%, product enantiomeric purity dropped from 94% *ee*_p_ to 85% *ee*_p_, and substrate enantiomeric excess decreased from 64% *ee*_s_ to 16% *ee*_s_ (**entry 7,** Table 3). Despite the lower conversion, the process remained viable since 10 < *E* < 30, allowing for the reuse of the immobilized enzyme. Spheres (**Hx2.0** and **Hx2.8**) outperformed mats (**Hx2.0** and mat‐layered structure **Hx2.0**), showing high *ee*_p_, and reasonable *E*, likely due to their shape facilitating a more efficient interaction with the substrate. The recycled **Hx2.8** spheres showed improved *ee*_s_ and *ee*_p_ over time, indicating that immobilized enzymes may regain efficiency. However, their E values were lower, reflecting decreased product enantiomeric purity.

Overall, enzymatic activity and product formation vary significantly depending on the different enzyme immobilization methods. The free PFL provided the highest efficiency, while among the immobilized samples, the spherical forms (**Hx2.0** and **Hx2.8**) performed better. Time plays a critical role in enzyme effectiveness, and while recycling immobilized enzymes is promising, it comes at the cost of reduced enantiomeric purity in subsequent cycles. Cellulose esters hold promise as sustainable platforms for enzyme immobilization; however, further research is necessary to optimize the process and enhance performance.

## 4 Conclusions

This study successfully demonstrated the esterification of cotton linter cellulose using a LiCl/DMAc solvent system with hexanoic anhydride and benzoyl chloride as esterifying agents. Cellulose esters were synthesized with degrees of substitution of 1, 2, and 3, and their structural integrity was confirmed via FTIR and 1H NMR. These derivatives were used to prepare electrospun mats, spheres, and mat‐layered structures. Electrospinning was conducted under optimized conditions, including flow rates of 5.5–45.5 µL min^−1^, needle‐collector distance of 10 cm, voltages of 15–25 kV, and solution concentrations of 9–11 wt.%. These parameters enabled the formation of ultrafine fibers with diameters as small as 150 nm. However, a significant presence of beads was observed in the fiber mats, as revealed by SEM analysis, highlighting the challenges in achieving uniform morphology.

Evaluating these cellulose esters in different forms as enzyme immobilization platforms revealed variable performance. While immobilization on electrospun mats showed negligible catalytic activity, likely due to enzyme inactivation or impaired access, the mat‐layered structures significantly improved catalytic performance, doubling the conversion rate compared to the **Hx2.0** mat. Spheres of **Hx2.8** prepared with 15% *Pseudomonas fluorescens* lipase exhibited superior performance, achieving 40% conversion after 120 h and 94% enantiomeric purity. Recycling experiments demonstrated a 24% reduction in conversion efficiency.

These findings highlight the potential of cellulose‐derived materials, particularly **Hx2.8** spheres, for biocatalytic applications. This study underscores the promise of cellulose esters in developing sustainable and high‐performance enzyme immobilization platforms. Future research should optimize material properties and immobilization strategies to expand their applications in materials science and biotechnology.

This research is believed to be the first to explore the potential of cellulose esters in creating platforms for the enzymatic immobilization of PFL to resolve a racemic mixture. The findings may lead to using PFL‐incorporated mat‐layered or spherical structures in biocatalysis for purposes beyond those outlined in this study. Additionally, the materials developed may be tested to support the immobilization of various enzymes. All potential applications are unequivocally directed toward sustainable and environmentally sound solutions.

## Conflicts of Interest

The authors declare no conflicts of interest.

## Supporting information




**Supporting file 1**: mabi70052‐sup‐0001‐SuppMat.docx

## Data Availability

The data that support the findings of this study are available from the corresponding author upon reasonable request.

## References

[1] S. Liu and J. Wang , “Exploring the Potential of Cellulose Benzoate Adsorbents Modified with Carbon Nanotubes and Magnetic Carbon Nanotubes for Microplastic Removal from Water,” Chemical Engineering Journal 469 (2023): 143910.

[2] X. an , Q. Lv , G. Dong , et al., “Insight Into the Preparation and Improved Properties of Cellulose Citrate Ester Hydrogel Slow‐Release Fertilizer,” Industrial Crops and Products 222 (2024): 119517.

[3] Q. Wang , Y. Li , D.‐W. Sun , and Z. Zhuu , “Enhancing Food Processing by Pulsed and High Voltage Electric Fields: Principles and Applications,” Critical Reviews in Food Science and Nutrition 58 (2018): 2285–2298. 29393667 10.1080/10408398.2018.1434609

[4] D. M. F. Sandrini , D. L. Morgado , A. J. A. de Oliveira , D. A. de Moraes , L. C. Varanda , and E. Frollini , “Cellulose Esters: Synthesis for Further Formation of Films With Magnetite Nanoparticles Incorporated,” International Journal of Biological Macromolecules 264 (2024): 130594. 38437931 10.1016/j.ijbiomac.2024.130594

[5] B. V. M. Rodrigues , R. T. Polez , O. A. E. Seoud , and E. Frollini , “Cellulose Acylation in Homogeneous and Heterogeneous Media: Optimization of Reactions Conditions,” International Journal of Biological Macromolecules 243 (2023): 125256. 37295694 10.1016/j.ijbiomac.2023.125256

[6] C. L. McCormick and D. K. Lichatowich , “Homogeneous Solution Reactions of Cellulose, Chitin, and Other Polysaccharides to Produce Controlled‐Activity Pesticide Systems,” Journal of Polymer Science: Polymer Letters Edition 17 (1979): 479–484.

[7] U. Henniges , S. Schiehser , T. Rosenau , and A. Potthast , “Cellulose Solubility: Dissolution and Analysis of “Problematic” Cellulose Pulps in the Solvent System DMAc/LiCl,” (2010): 165–177.

[8] L. A. Ramos , J. M. Assaf , O. A. E. Seoud , and E. Frollini , “Influence of the Supramolecular Structure and Physicochemical Properties of Cellulose on Its Dissolution in a Lithium Chloride/N,N‐Dimethylacetamide Solvent System,” Biomacromolecules 6 (2005): 2638–2647. 16153102 10.1021/bm0400776

[9] Y. Hu , V. D. Thalangamaarachchige , S. Acharya , and N. Abidi , “Role of Low‐Concentration Acetic Acid in Promoting Cellulose Dissolution,” Cellulose 25 (2018): 4389–4405.

[10] V. Raus , A. Šturcová , J. Dybal , et al., “Activation of Cellulose by 1,4‐Dioxane for dissolution in N,N‐dimethylacetamide/LiCl,” Cellulose 19 (2012): 1893–‐1906.

[11] F. Şahin , N. Kayra , and A. Ö. Aytekin , “Optimizing the Production of Bacterial Cellulose Nanofibers and Nanocrystals Through Strategic Fiber Pretreatment,” Biopolymers 116 (2025). 10.1002/bip.23634 PMC11661429 39360416

[12] A. J. Sayyed , N. A. Deshmukh , and D. V. Pinjari , “A Critical Review of Manufacturing Processes Used in Regenerated Cellulosic Fibres: Viscose, Cellulose Acetate, Cuprammonium, LiCl/DMAc, Ionic Liquids, and NMMO Based Lyocell,” Cellulose 26 (2019): 2913–2940.

[13] Y. Zhao , X. Wang , B. Li , et al., “Activation Behavior of 4‐AcNH‐TEMPO by ClO2 for Selective Oxidation of Cellulose in DMAc/LiCl system,” Cellulose 32 (2025): 3007–3026.

[14] A. Turbak , “Other Processes,” in Regenerated Cellulose Fibres, ed. C Woodings (CRC Press, 2001), 174–198.

[15] M. Kostag , M. Gericke , T. Heinze , and O. A. E. Seoud , “Twenty‐Five Years of Cellulose Chemistry: Innovations in the Dissolution of the Biopolymer and its Transformation Into Esters and Ethers,” Cellulose 26 (2019): 139–184.

[16] Y.‐Y. Ma , Z.‐L. Lu , Y.‐Z. Xing , W.‐S. Zheng , and C.‐G. Liu , “A Fresh Perspective on Dissociation Mechanism of Cellulose in DMAc/LiCl System Based on Li Bond Theory,” International Journal of Biological Macromolecules 268 (2024): 131729. 38653429 10.1016/j.ijbiomac.2024.131729

[17] B. G. Queiroz , H. Ciol , N. M. Inada , and E. Frollini , “Cross‐Linked Bio‐Based Hydrogels Generated From Solutions Derived From the Deconstruction of Sisal Fibers,” Journal of Molecular Liquids 369 (2023): 120876.

[18] A. Ibaraki , S. Kaneta , and T. Kobayashi , “The Effect of Oxidative Bleaching Using Chlorine Oxoacid Agents on the Characteristics of Cellulose Fibers and Hydrogel Films of Sugarcane Bagasse,” Waste and Biomass Valorization 16 (2025): 319–331.

[19] Y. Zhang , K. Kobayashi , and M. Wada , “Comparative Analysis of the Structures and Properties of Cellulose Hydrogels Prepared Using Different Solvent Systems,” Cellulose 2 (2025): 2337–2351.

[20] L. Yang , J. Meng , T. Xue , et al., “Application of 3D Printing Cellulose Fabrics Based on Cotton Fibers in the Textile and Fashion Industry,” Additive Manufacturing 81 (2024): 104000.

[21] J. Liu , H. Zhang , X. Jiang , P.‐L. Tremblay , and T. Zhang , “An Efficient and Reusable N,N‐dimethylacetamide/LiCl Solvent System for the Extraction of High‐Purity Polyhydroxybutyrate from Bacterial Biomass,” Biochemical Engineering Journal 192 (2023): 108812.

[22] H. Liu and Y.‐L. Hsieh , “Ultrafine Fibrous Cellulose Membranes From Electrospinning of Cellulose Acetate,” Journal of Polymer Science Part B: Polymer Physics 40 (2002): 2119–2129.

[23] T. Ferracini R. Voltarelli P. Passos de Oliveira Santos F. Rossi , and E. Frollini , “Exploring the Formation of Cellulose Acetate Mats From Electrospinning of Dimethylacetamide/Tetrahydrofuran Solutions,” Biomass Conversion and Biorefinery 15 (2024): 13997–14015.

[24] J. Kerwald and C. F. M. Junior , “Cellulose‐Based Electrospun Nanofibers: A Review,” Cellulose 29 (2022): 25–54.

[25] S. Rahmani , Z. Khoubi‐Arani , S. Mohammadzadeh‐Komuleh , and M. Maroufkhani , “Electrospinning of Cellulose Nanofibers for Advanced Applications,” Handbook of Nanocelluloses (Springer International Publishing, 2021), 1–34.

[26] R. Clementi , M. A. Vargas , M. Cid , N. Salvatierra , R. Comín , and T. Tempesti , “Biocompatible Zn‐Phthalocyanine/Gelatin Nanofiber Membrane for Antibacterial Therapy,” Macromolecular Bioscience 25 (2025). 10.1002/mabi.202400334 39470704

[27] S. Nono‐Tagne , T. Heinze , M. Gericke , and I. Otsuka , “Electrospinning of Cellulose Benzyl Carbamates for Enantioselective Membrane Filtration,” Macromolecular Bioscience 25 (2024). 10.1002/mabi.202400415 PMC11904391 39601524

[28] Y. Zhang , C. Zhang , and Y. Wang , “Recent Progress in Cellulose‐Based Electrospun Nanofibers as Multifunctional Materials,” Nanoscale Advances 3 (2021): 6040–6047. 36133945 10.1039/d1na00508a PMC9417631

[29] C. Zhu and J. Zheng , and J. Fu , “Electrospinning Nanofibers as Stretchable Sensors for Wearable Devices,” Macromolecular Bioscience 24 (2024). 10.1002/mabi.202300274 37653597

[30] Y. Liu and J. Y. Chen , “Enzyme Immobilization on Cellulose Matrixes,” Journal of Bioactive and Compatible Polymers 31 (2016): 553–567.

[31] Q. Luan , H. Zhang , Y. Lei , et al., “Microporous Regenerated Cellulose‐Based Macrogels for Covalent Immobilization of Enzymes,” Cellulose 28 (2021): 5735–5744.

[32] S. Sulaiman , M. Noriznan Mokhtar , M. Nazli Naim , A. Samsu Baharuddin , and A. Sulaiman , “A Review: Potential Usage of Cellulose Nanofibers (CNF) for Enzyme Immobilization via Covalent Interactions,” Applied Biochemistry and Biotechnology 175 (2015): 1817–1842. 25427594 10.1007/s12010-014-1417-x

[33] D. Murtinho , A. R. Lagoa , F. A. P. Garcia , and M. H. Gil , “Cellulose Derivatives Membranes as Supports for Immobilisation of Enzymes,” Cellulose 5 (1998): 299–308.

[34] J. P. F. Carvalho , A. C. Q. Silva , A. J. D. Silvestre , C. S. R. Freire , and C. Vilela , “Spherical Cellulose Micro and Nanoparticles: A Review of Recent Developments and Applications,” Nanomaterials 11 (2021): 2744. 34685185 10.3390/nano11102744 PMC8537411

[35] J. Chapman , A. E. Ismail , and C. Z. Dinu , “Industrial Applications of Enzymes: Recent Advances, Techniques, and Outlooks,” Catalysts 8 (2018): 238.

[36] T. Dinmukhamed , Z. Huang , Y. Liu , et al., “Current Advances in Design and Engineering Strategies of Industrial Enzymes,” Systems Microbiology and Biomanufacturing 1 (2021): 15–23.

[37] A. Carvalho , T. Fonseca , M. Mattos , et al., “Recent Advances in Lipase‐Mediated Preparation of Pharmaceuticals and Their Intermediates,” International Journal of Molecular Sciences 16 (2015): 29682–29716. 26690428 10.3390/ijms161226191 PMC4691134

[38] F. Golombek , M. Haumann , M. S. G. Knoll , A. Paul Fröba , and K. Castiglione , “Three Steps, Two Enzymes, One Pot, but a Multitude of Nanocompartments: Combined Cycles of Kinetic Resolutions and Re‐Racemization With Incompatible Biocatalysts,” ACS Omega 6 (2021): 29192–29200. 34746608 10.1021/acsomega.1c04694 PMC8567398

[39] F. Passannanti , M. Gallo , G. Lentini , et al., “Alginate Capsules: Versatile Applications and Production Techniques,” Macromolecular Bioscience 24 (2024). 10.1002/mabi.202400202 39233662

[40] M. H. Kim , S. An , K. Won , H. J. Kim , and S. H. Lee , “Entrapment of Enzymes Into Cellulose–Biopolymer Composite Hydrogel Beads Using Biocompatible Ionic Liquid,” Journal of Molecular Catalysis B: Enzymatic 75 (2012): 68–72.

[41] B. A. P. Ass , M. N. Belgacem , and E. Frollini , “Mercerized Linters Cellulose: Characterization and Acetylation in N,N‐Dimethylacetamide/Lithium Chloride in N,N‐Dimethylacetamide/Lithium Chloride,” Carbohydrate Polymers 63 (2006): 19–29.

[42] O. A. E. Seoud , L. C. Fidale , N. Ruiz , M. L. O. D'Almeida , and E. Frollini , “Cellulose Swelling by Protic Solvents: Which Properties of the Biopolymer and the Solvent Matter?,” Cellulose 15 (2008): 371–392.

[43] L. Segal , J. J. Creely , A. E. Martin , and C. M. Conrad , “An Empirical Method for Estimating the Degree of Crystallinity of Native Cellulose Using the X‐Ray Diffractometer,” Textile Research Journal 29 (1959): 786–794.

[44] E. V. R. Almeida , D. L. Morgado , L. A. Ramos , and E. Frollini , “Sisal Cellulose and Its Acetates: Generation of Films and Reinforcement in a One‐Pot Process,” Cellulose 20 (2013): 453–465.

[45] R. T. Polez , B. V. M. Rodrigues , O. A. E. Seoud , and E. Frollini , “Electrospinning of Cellulose Carboxylic Esters Synthesized Under Homogeneous Conditions: Effects of the Ester Degree of Substitution and Acyl Group Chain Length on the Morphology of the Fabricated Mats,” Journal of Molecular Liquids 339 (2021): 116745.

[46] B. V. M. Rodrigues , R. T. Polez , O. A. El Seoud , and E. Frollini , “Cellulose Acylation in Homogeneous and Heterogeneous Media: Optimization of Reactions Conditions,” International Journal of Biological Macromolecules 243 (2023): 125256. 37295694 10.1016/j.ijbiomac.2023.125256

[47] V. W. Goodlett , J. T. Dougherty , and H. W. Patton , “Characterization of Cellulose Acetates by Nuclear Magnetic Resonance,” Journal of Polymer Science Part A‐1: Polymer Chemistry 9 (1971): 155–161.

[48] B. A. P. Ass , M. N. Belgacem , and E. Frollini , “Mercerized Linters Cellulose: Characterization and Acetylation in N,N‐Dimethylacetamide/Lithium Chloride,” Carbohydrate Polymers 63 (2006): 19–29.

[49] J. Chen , J. Xu , K. Wang , X. Cao , and R. Sun , “Cellulose Acetate Fibers Prepared From Different Raw Materials with Rapid Synthesis Method,” Carbohydrate Polymers 137 (2016): 685–692. 26686180 10.1016/j.carbpol.2015.11.034

[50] V. W. Goodlett , J. T. Dougherty , and H. W. Patton , “Characterization of Cellulose Acetates by Nuclear Magnetic Resonance,” Journal of Polymer Science Part A‐1: Polymer Chemistry 9 (1971) 155–161.

[51] J. Zhang , J. Wu , Y. Cao , S. Sang , J. Zhang , and J. He , “Synthesis of Cellulose Benzoates Under Homogeneous Conditions in an Ionic Liquid,” Cellulose 16 (2009): 299–308.

[52] B. V. M. Rodrigues , E. C. Ramires , R. P. O. Santos , and E. Frollini , “Ultrathin and Nanofibers via Room Temperature Electrospinning From Trifluoroacetic Acid Solutions of Untreated Lignocellulosic Sisal Fiber or Sisal Pulp,” Journal of Applied Polymer Science 132 (2015).

[53] S. S. Ribeiro , C. Raminelli , and A. L. M. Porto , “Enzymatic Resolution by CALB of Organofluorine Compounds Under Conventional Condition and Microwave Irradiation,” Journal of Fluorine Chemistry 154 (2013): 53–59.

[54] L. C. Rocha , I. G. Rosset , R. F. Luiz , C. Raminelli , and A. L. M. Porto , “Kinetic Resolution of Iodophenylethanols by Candida Antarctica Lipase and Their Application for the Synthesis of Chiral Biphenyl Compounds,” Tetrahedron: Asymmetry 21 (2010): 926–929.

[55] C. Chen , Y. Fujimoto , G. Girdaukas , and C. J. Sih , “Quantitative Analyses of Biochemical Kinetic Resolutions of Enantiomers,” Journal of the American Chemical Society 104 (1982): 7294–7299.

[56] V. E. U. Costa and H. L. N. Amorim , “O emprego De Lipases Como Agentes de Resolução Cinética de Enantiômeros em Síntese Orgânica: Aspectos Gerais Sobre a Influência do Solvente,” Química Nova 22 (1999): 863–873.

[57] R. T. Polez , B. V. M. Rodrigues , O. A. El Seoud , and E. Frollini , “Electrospinning of Cellulose Carboxylic Esters Synthesized Under Homogeneous Conditions: Effects of the Ester Degree of Substitution and Acyl Group Chain Length on the Morphology of the Fabricated Mats,” Journal of Molecular Liquids 339 (2021): 116745.

[58] B. V. M. Rodrigues , E. C. Ramires , R. P. O. Santos , and E. Frollini , “Ultrathin and Nanofibers via Room Temperature Electrospinning From Trifluoroacetic Acid Solutions of Untreated Lignocellulosic Sisal Fiber or Sisal Pulp,” Journal of Applied Polymer Science 132 (2015).

[59] Y. Luan , J. Zhang , M. Zhan , J. Wu , J. Zhang , and J. He , “Highly Efficient Propionylation and Butyralation of Cellulose in an Ionic Liquid Catalyzed by 4‐Dimethylminopyridine,” Carbohydrate Polymers 92 (2013): 307–311. 23218299 10.1016/j.carbpol.2012.08.111

[60] L. Chang , J. Zhang , W. Chen , et al., “Controllable Synthesis of Cellulose Benzoates for Understanding of Chiral Recognition Mechanism and Fabrication of Highly Efficient Chiral Stationary Phases,” Analytical Methods 10 (2018): 2844–2853.

[61] Y. Kim , D. Jeong , K. Hui Park , J.‐H. Yu , and S. Jung , “Efficient Adsorption on Benzoyl and Stearoyl Cellulose to Remove Phenanthrene and Pyrene From Aqueous Solution,” Polymers 10 (2018): 1042. 30960967 10.3390/polym10091042 PMC6403814

[62] N. Angel , L. Guo , F. Yan , H. Wang , and L. Kong , “Effect of Processing Parameters on the Electrospinning of Cellulose Acetate Studied by Response Surface Methodology,” Journal of Agriculture and Food Research 2 (2020): 100015.

[63] R. Korehei , J. Olson , F. Ko , and J. Kadla , “Influence of the Solvent and Nonsolvent Composition on the Electrospinning of a Cellulose Acetate Ternary System,” Journal of Applied Polymer Science 132 (2015).

[64] M. Hasegawa , A. Isogai , F. Onabe , and M. Usuda , “Dissolving States of Cellulose and Chitosan in Trifluoroacetic Acid,” Journal of Applied Polymer Science 45 (1992): 1857–1863.

[65] K. Ohkawa , S. Hayashi , A. Nishida , H. Yamamoto , and J. Ducreux , “Preparation of Pure Cellulose Nanofiber via Electrospinning,” Textile Research Journal 79 (2009): 1396–1401.

[66] R. P. O. Santos , B. V. M. Rodrigues , D. M. Santos , S. P. Campana‐Filho , A. C. Ruvolo‐Filho , and E. Frollini , “Electrospun Recycled PET‐Based Mats: Tuning the Properties by Addition of Cellulose and/or Lignin,” Polymer Testing 60 (2017): 422–431.

[67] H. Fong , I. Chun , and D. H. Reneker , “Beaded Nanofibers Formed During Electrospinning,” Polymer 40 (1999): 4585–4592.

[68] A. Bonakdar , Mahboubeh , and D. Rodrigue , “Electrospinning: Processes, Structures, and Materials,” Macromol 4 (2024): 58–103.

[69] M. S. Islam , B. C. Ang , A. Andriyana , and A. M. Afifi , “A Review on Fabrication of Nanofibers via Electrospinning and Their Applications,” SN Applied Sciences 1 (2019): 1248.

[70] J. Xue , T. Wu , Y. Dai , and Y. Xia , “Electrospinning and Electrospun Nanofibers: Methods, Materials, and Applications,” Chemical Reviews 119 (2019): 5298–5415. 30916938 10.1021/acs.chemrev.8b00593 PMC6589095

[71] M. M. Demir , I. Yilgor , E. Yilgor , and B. Erman , “Electrospinning of Polyurethane Fibers,” Polymer 43 (2002): 3303–3309.

[72] E. Goli , S. R. Peterson , and P. H. Geubelle , “Instabilities Driven by Frontal Polymerization in Thermosetting Polymers and Composites,” Composites Part B: Engineering 199 (2020): 108306.

[73] I. Greenfeld , C. W. Rodricks , X. M. Sui , and H. Daniel Wagner , “Beaded Fiber Composites—Stiffness and Strength Modeling,” Journal of the Mechanics and Physics of Solids 125 (2019): 384–400.

[74] T. Lu , J. Cui , Q. Qu , et al., “Multistructured Electrospun Nanofibers for Air Filtration: A Review,” ACS Applied Materials & Interfaces 13 (2021): 23293–23313. 33974391 10.1021/acsami.1c06520

[75] J. Bras , C. Vaca‐Garcia , M.‐E. Borredon , and W. Glasser , “Oxygen and Water Vapor Permeability of Fully Substituted Long Chain Cellulose Esters (LCCE),” Cellulose 14 (2007): 367–374.

[76] Y. Wang , X. Wang , Y. Xie , and K. Zhang , “Functional Nanomaterials Through Esterification of Cellulose: A Review of Chemistry and Application,” Cellulose 25 (2018): 3703–3731.

[77] K. Faber , “Biocatalytic Applications,” Biotransformations in Organic Chemistry (Springer International Publishing, 2018), 31–313.

[78] A. C. O. Machado , A. A. T. Silva , C. P. Borges , A. B. C. Simas , and D. M. G. Freire , “Kinetic Resolution of (R,S)‐1,2‐Isopropylidene Glycerol (Solketal) Ester Derivatives by Lipases,” Journal of Molecular Catalysis B: Enzymatic 69 (2011): 42–46.

[79] M. M. Poojary , S. Roohinejad , M. Koubaa , et al., “Impact of Pulsed Electric Fields on Enzymes,” Handbook of Electroporation (Springer International Publishing, 2017), 2369–2389.

[80] D. Niu , X.‐A. Zeng , E.‐F. Ren , et al., “Review of the Application of Pulsed Electric Fields (PEF) Technology for Food Processing in China,” Food Research International 137 (2020): 109715. 33233287 10.1016/j.foodres.2020.109715

[81] S. Ho and G. S. Mittal , “High Voltage Pulsed Electrical Field for Liquid Food Pasteurization,” Food Reviews International 16 (2000): 395–434.

[82] S. Bendicho , A. Espachs , J. Arántegui , and O. Martín , “Effect of High Intensity Pulsed Electric Fields and Heat Treatments on Vitamins of Milk,” Journal of Dairy Research 69 (2002): 113–123. 12047102 10.1017/s0022029901005258

[83] M. M. Aldhahri , Y. Q. Almulaiky , R. M. El‐Shishtawy , W. Al‐Shawafi , A. Alngadh , and R. Maghrabi , “Facile Immobilization of Enzyme via Co‐Electrospinning: A Simple Method for Enhancing Enzyme Reusability and Monitoring an Activity‐Based Organic Semiconductor,” ACS Omega 3 (2018): 6346–6350. 31458817 10.1021/acsomega.8b00366 PMC6644564

[84] H. Zhao , A. Roy , A. Samaranayake , et al., “Lipase‐Catalyzed Michael Addition in ‘Water‐Like’ Ionic Liquids and Tertiary Amides: What Is the Role of the Enzymes?” Langmuir 41 (2025): 12718–12730. 40356070 10.1021/acs.langmuir.5c00874

[85] A. Kumar , K. Dhar , S. Singh Kanwar , and P. K. Arora , “Lipase Catalysis in Organic Solvents: Advantages and Applications,” Biological Procedures Online 18 (2016): 2. 26766927 10.1186/s12575-016-0033-2 PMC4711063

[86] I. M. Ferreira , R. H. V. Nishimura , A. B. A. Souza , G. C. Clososki , S. A. Yoshioka , and A. L. M. Porto , “Highly Enantioselective Acylation of Chlorohydrins Using Amano AK Lipase From P. Fluorescens Immobilized on Silk Fibroin–Alginate Spheres,” Tetrahedron Letters 55 (2014): 5062–5065.

[87] I. M. Ferreira , S. A. Yoshioka , J. V. Comasseto , and A. L. M. Porto , “Immobilization of Amano Lipase From Pseudomonas Fluorescens on Silk Fibroin Spheres: An Alternative Protocol for the Enantioselective Synthesis of Halohydrins,” RSC Advances 7 (2017): 12650–12658.

