# Supplementary material for: Synthesis of Cellulose Hexanoate, Benzoate, and Mixed Esters: Exploring Their Potential as Enzyme Immobilization Platforms
Source: Macromol Biosci. 2025 Aug 6;25(11):e00221. doi: 10.1002/mabi.202500221 (PMC12617693; doi:10.1002/mabi.202500221)
Supplement: Supplementary file 1 — Supporting file 1: mabi70052‐sup‐0001‐SuppMat.docx [file MABI-25-e00221-s001.docx]

**Supporting Information**

**Synthesis of Cellulose Hexanoate, Benzoate and Mixed Esters: Exploring Their Potential as Enzyme Immobilization Platforms**

*Roberta Teixeira Polez, Thamiris Voltarelli Ferracini, Samuel Filipe Cardoso de Paula, Rachel Passos de Oliveira Santos, André Luiz Meleiro Porto, Elisabete Frollini**

[*elisabete@iqsc.usp.br](mailto:*elisabete@iqsc.usp.br)

**Table S1.** Residual quantity of the elements C, H, and N in the cellulose esters determined through elemental analysis and residual quantity of lithium in the cellulose esters determined via atomic absorption spectrophotometry.

| **Ester code** | **C (%)** | **H (%)** | **N (%)** | **Li (ppb)** |
| --- | --- | --- | --- | --- |
| **Hx1.0** | 54.3 ± 1.2 | 6.7 ± 0.06 | N.D.* | N.D. |
| **Hx2.0** | 58.1 ± 1.9 | 8.1 ± 0.2 | N.D. | N.D. |
| **Hx2.8** | 57.6 ± 2.4 | 6.9 ± 0.3 | N.D. | N.D. |
| **Bz0.7** | 53.2 ± 0.4 | 6.0 ± 0.07 | N.D. | 16.4 ± 0.8 |
| **Bz1.3** | 64.6 ± 0.4 | 5.1 ± 0.03 | 0.11 ± 0.02 | 190.9 ± 0.9 |
| **Bz1.6** | 63.9 ± 0.9 | 4.9 ± 0.06 | 0.08 ± 0.02 | 56.8 ± 1.4 |
| **HxBz1/1** | 60.6 ± 1.8 | 8.3 ± 0.2 | 0.06 ± 0.02 | 222 ± 0.9 |
| **HxBz0.9/0.3** | 53.6 ± 0.4 | 7.0 ± 0.05 | 0.11 ± 0.09 | 203 ± 1.1 |

* Non Detected


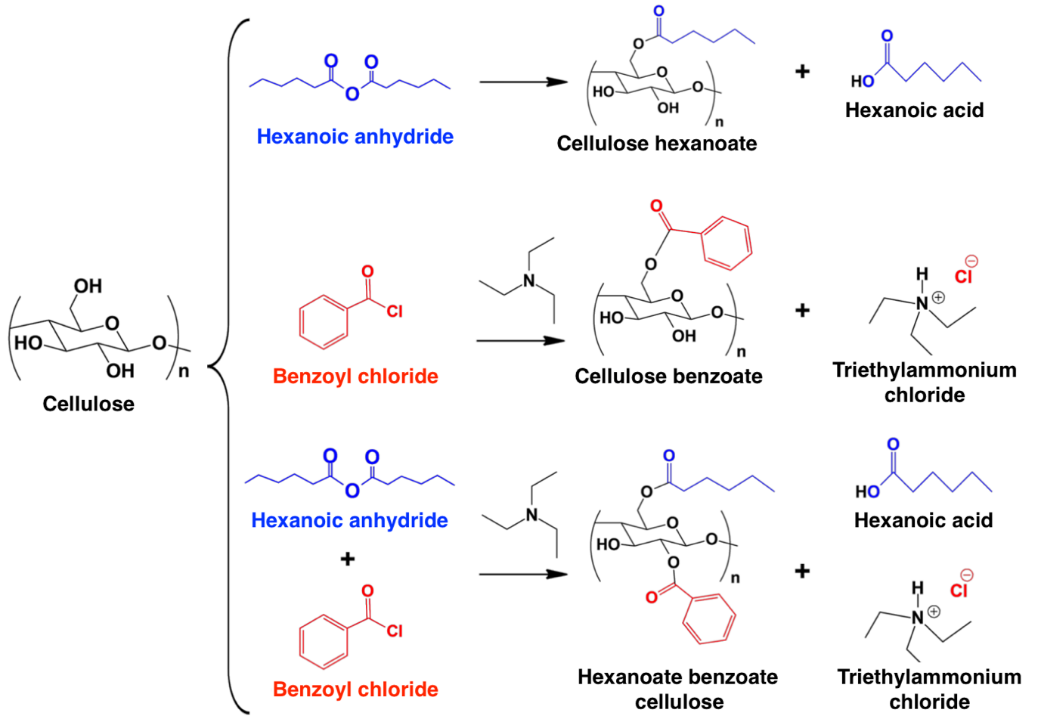


**Figure S1.** Esterification reactions of cellulose.


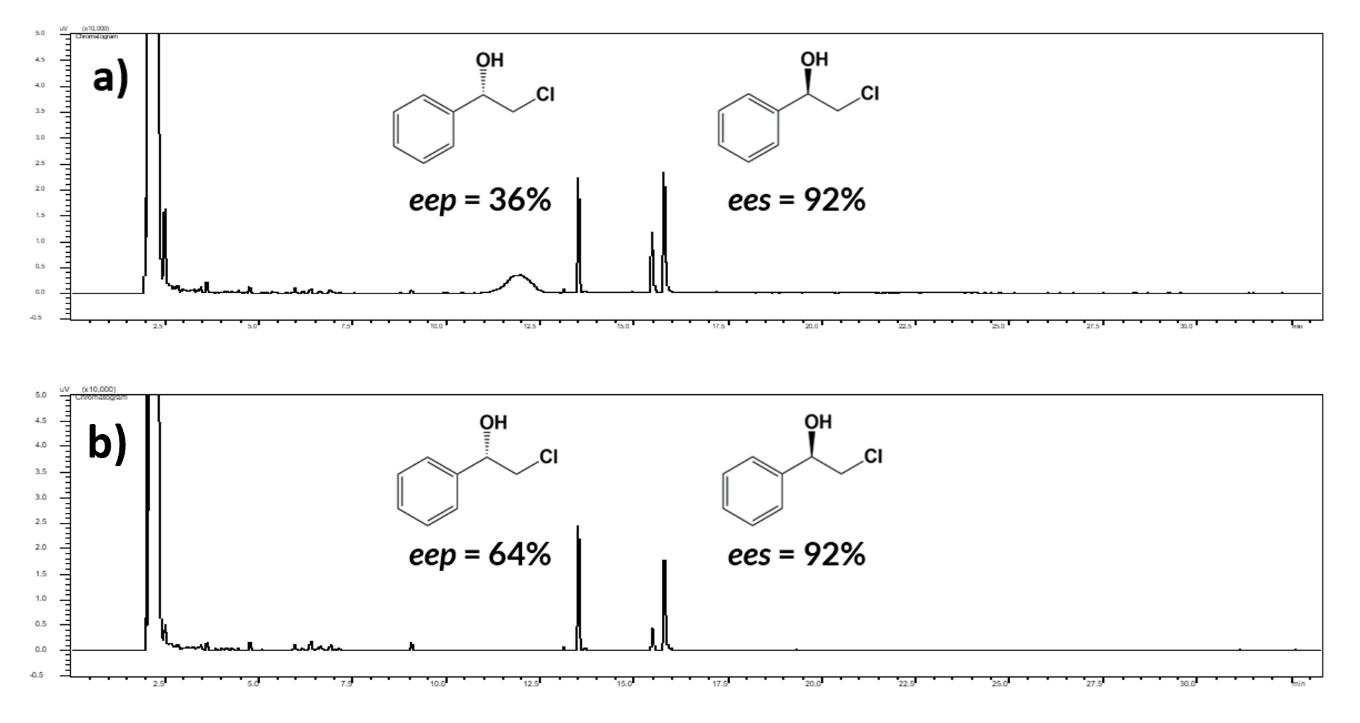


**Figure S2.** Chromatograms obtained by GC-FID of the enzymatic kinetic resolution of (R,S)-2-chloro-1-phenylethan-1-ol) using lipase *Pseudomonas fluorescens* immobilized on spheres: **(a)** **Hx2.0** after 120 h and **(b) Hx2.8** after 120 h. ee_s_ and ee_p_ represent the enantiomeric excess of the substrate and product, respectively.
